# Multi-Model Adaptation Learning With Possibilistic Clustering Assumption for EEG-Based Emotion Recognition

**DOI:** 10.3389/fnins.2022.855421

**Published:** 2022-05-04

**Authors:** Yufang Dan, Jianwen Tao, Di Zhou

**Affiliations:** ^1^Institute of Artificial Intelligence Application, Ningbo Polytechnic, Ningbo, China; ^2^Key Laboratory of 3D Printing Equipment and Manufacturing in Colleges and Universities of Fujian Province, Fujian, China; ^3^Industrial Technological Institute of Intelligent Manufacturing, Sichuan University of Arts and Science, Dazhou, China

**Keywords:** semi-supervised learning, multi-model adaptation, clustering assumption, encephalogram, fuzzy entropy, emotion recognition

## Abstract

In machine learning community, graph-based semi-supervised learning (GSSL) approaches have attracted more extensive research due to their elegant mathematical formulation and good performance. However, one of the reasons affecting the performance of the GSSL method is that the training data and test data need to be independently identically distributed (IID); any individual user may show a completely different encephalogram (EEG) data in the same situation. The EEG data may be non-IID. In addition, noise/outlier sensitiveness still exist in GSSL approaches. To these ends, we propose in this paper a novel clustering method based on structure risk minimization model, called multi-model adaptation learning with possibilistic clustering assumption for EEG-based emotion recognition (MA-PCA). It can effectively minimize the influence from the noise/outlier samples based on different EEG-based data distribution in some reproduced kernel Hilbert space. Our main ideas are as follows: (1) reducing the negative impact of noise/outlier patterns through fuzzy entropy regularization, (2) considering the training data and test data are IID and non-IID to obtain a better performance by multi-model adaptation learning, and (3) the algorithm implementation and convergence theorem are also given. A large number of experiments and deep analysis on real DEAP datasets and SEED datasets was carried out. The results show that the MA-PCA method has superior or comparable robustness and generalization performance to EEG-based emotion recognition.

## Introduction

Emotion is a psychological experience from human beings of the world, which is complex and changeable (Dolan, 2002; Zhang et al., 2016, 2019b). Different human beings have different emotional understanding on the same thing and may make misjudgment about the emotion occasionally, let alone machines. Therefore, emotion recognition has attracted great attention from researchers (Kim et al., 2013; Mühl et al., 2014; Zhao et al., 2015, 2016; Chu et al., 2017). In this paper, we mainly recognize the corresponding emotion by the internal changes of the human body which include the heart rate, blood pressure, respiratory rate, magneto encephalogram, electroencephalogram (Mühl et al., 2014), and so on. Generally, most existing EEG-based emotion recognition systems are divided into two steps: data preparation and classifier training (Lan et al., 2018; Zhang et al., 2020). EEG feature extraction methods are comprehensively sorted out in Jenke et al. (2014). In order to improve the recognition accuracy, there existed many EEG-based emotion recognition approaches (Musha et al., 1997; Kim et al., 2013). A satisfactory state of emotion detection based on brain computer interface (BCI) is to detect the emotional state by a real-time EEG signal without inputting signal from the subjects (Zhang et al., 2019b). Different feedback is given to different emotional states in the meantime. These proposed methods (Zhang et al., 2016, 2017) are used to recognize multiple emotion classes from EEG. The latest affective BCIs adopted machine learning algorithms and relied on a few support vectors (Jenke et al., 2014; Mühl et al., 2014). For recording of the EEG signal of the expected target emotion, it is necessary to provide emotional stimulation of the expected concrete emotion to subjects. In the training/calibration stage, the EEG datasets with labels are used to train the emotion recognizer. Many researchers have reported sound classification performance on emotion recognition from real-time EEG data (Mühl et al., 2014).

Due to the high cost of obtaining labeled data, semi-supervised learning (SSL) technology has appeared. It only needs a small, labeled data and a large, unlabeled data to learn a model, which solves the problem on supervised learning needing a large number of labeled samples. Tu and Sun (2013) presented an EEG classifier *via* SSL feature extraction strategy. Tao et al. (2015, 2016, 2017) and Wu and Deng (2018) showed a SSL method for reducing the possible negative impact from random initialization parameters on neural networks. Zu et al. (2019) proposed that remote sensing image classification method based on SSL can effectively improve the accuracy of land cover classification and has a higher efficiency in remote sensing image classification since graph-based semi-supervised learning (GSSL) (Li and Zhou, 2011; Liu et al., 2012; Wang et al., 2012), with its good performance, has been extensively studied. The manifold regularization (MR) (Belkin et al., 2006; Gao et al., 2010; Nie et al., 2010) is a popular GSSL method. A general MR framework was presented by Nie et al. (2010).

In general, the clustering assumption is a basic assumption in GSSL: similar samples should belong to the same class (Chapelle, 2006; Zhu and Goldberg, 2009; Xue et al., 2011; Zhou et al., 2014; Wang et al., 2019). In other words, each sample only have one label, which we called hard classification. However, in the real applications of emotion recognition, its performance will be discounted by this assumption—for example, for different subjects in different scenes, crying may be understood as sad and happy.

To handle the limitation from this assumption, Wang et al. (2012) and Zhang et al. (2019b) proposed a novel clustering assumption that can significantly boost the classifier performance. Assuming that similar samples have the same label membership, each sample may have multiple membership values (Zhang et al., 2019a), not only one. However, this method has a constraint in that the sum of the membership values of each sample is 1. This constraint may lead to the membership values of some noise being close to or even greater than those of normal samples; it may lead to misrecognition.

According to this problem in the SSCCM method, Dan et al. (2021) proposed SSPCA that relaxed the constraint in SSCCM and added a fuzzy entropy regularization term (Kosko, 1986; Krishnapuram and Keller, 1993; Zhang et al., 2019c) for increasing the samples’ discriminative information to get a membership function with better generalization and that can also reduce the negative impact of noise and outlier on recognition performance to improve the robustness of the method. Wang and Chen (2013) designed SA-SSCCM. Specifically, the SSCCM method is upper boundary and the LS-SVM method is lower boundary, respectively—that is, if unlabeled data is good for model training, the classification result of SA-SSCCM is close to SSCCM; if unlabeled data penalizes model training, the classification result of SA-SSCCM is close to that of the LS-SVM method, and the interference of noise data to the SA-SSCCM model training is avoided. However, both SSPCA method and SA-SSCCM method require training data, and the test data should be independently identically distributed (IID). Due to the difference among different subjects in real emotion recognition applications, it may cut recognition accuracy. It is hard to guarantee that two datasets are IID.

Toward the problem of reduced recognition accuracy caused by non-IID of training data and test data, this paper adopts domain adaptation learning (DAL) (Bruzzone and Marconcini, 2010; Tao et al., 2021) related to computer vision and machine learning (Bishop, 2006; Zhu, 2008). Generally, DAL includes instance-based DAL, feature-based DAL, and model-based DAL (Pan and Yang, 2010). The instance-based DAL and feature-based DAL need to access instances from the source domain during the model learning. When the source dataset is relatively large, the training efficiency will be reduced. The model-based DAL uses the pre-trained source classifier on some source datasets to learn an effective target classifier, which has good classification effectiveness and high efficiency. Much more DAL categories in Pan and Yang (2010) can be found. Therefore, this paper proposes a multi-model adaptation learning with possibilistic clustering assumption for EEG-based emotion recognition (MA-PCA).

The main ideas are as follows: firstly, according to manifold learning (Tenenbaum et al., 2000; Belkin and Niyogi, 2001; Gao et al., 2010; Nie et al., 2010), there is similarity among samples within its local. According to formula (1) in Dan et al. (2021), the local weighted mean (LWM) point is determined by the convex hull of the k-nearest neighbors. It represents the mean value of the local. Therefore, the neighbors in the local should have consistency with the mean value—that is, the labels of each neighbor in the local and its corresponding LWM should be similar (or consistent). Then, it is assumed that each neighbor has a similar label membership to its corresponding LWM (Bottou and Vapnik, 1992; Atkeson et al., 1997; Xue and Chen, 2007); secondly, the classification prediction results are mutually verified by the decision function and the membership function to improve the classification reliability; thirdly, a fuzzy entropy regularization term is proposed to increase the sample discrimination information; then, we can get a membership function with better generalization, and the negative impact of noise and outlier will be relaxed on recognition performance. Finally, a classification model with better generalization performance is obtained by adding a multi-model adaptation regularization term for IID and non-IID on training data and test data, respectively. The major contributions of this work are the following:

(1) A multi-model adaptation learning with possibilistic clustering assumption for EEG-based (MA-PCA) is proposed.

(2) Since a multiple auxiliary discriminant model is good for SSL with a small labeled instance, the regular term has Laplacian local consistency and the regularize term has different weights of multiple-source models. It aims to expand the discriminant space of the target domain and guarantee the local structure consistency among inner samples of source domain and target domain. At the end, it solves the non-IID problem between the training data and test data, too.

(3) Finally, our comprehensive experiments on real datasets (i.e., DEAP, SEED) show that the method has better robustness and generalization.

The remainder of this work is organized as follows: In section “Proposed Framework,” our framework MA-PCA will be designed which includes MA-PCA formulation, optimization, and convergence analysis, and section “Algorithm of MA-PCA” arranges the corresponding optimal algorithm of MA-PCA. The experimental results and analysis on two real EEG datasets (i.e., DEAP and SEED) are presented in section “Experimental Evaluation.” Finally, we conclude in section “Conclusion.”

## MA-PCA Framework

This section will introduce the concept of our multi-model adaptation learning with possibilistic clustering assumption for EEG-based (MA-PCA) framework in detail. It mainly uses multiple-source models which are obtained from existing relevant source datasets to learn the robust semi-supervised classification model. Therefore, the two core components are organically unified into MA-PCA: (1) any instance should have a similar label membership with its corresponding LWM. The fuzzy entropy regularization term is added to reach the amount of discrimination information to improve the classification accuracy and robustness and (2) assuming that multiple-source models can help SLL, the existing multi-source models are used for multi-source domain adaptation learning to establish a robust target domain classification model. At the same time, considering IID and non-IID, the best source model is found by multiple-source models with different weights to train the target model.

### Notations

We denote *X* = {x_1_,x_2_,…,x*i*,_x*i* + 1_,…,_x*n*_} as a feature dataset, where *n* is sample number (*l*≪*n*) and *Y*_*l*_ = ^{*y*_1_,*y*_2_,…,*y*_*l*_}*T*^ ∈ ℝ*l*×*^M^* is a label set about dataset$X_{l}={\left\{x_{i}\right\}}_{i=1}^{l}$. $X_{u}={\left\{x_{j}\right\}}_{j=l+1}^{n}$ is an unlabeled feature dataset, where *x*_*i*_ is *d* dimensions (*x*_*i*_ ∈ ^*Rd*^) vector of the *i*-th sample. We compute LWM ${\hat{x}}_{i}$ about *x*_*i*_:


(1) $${\hat{x}}_{i}=\frac{\sum_{x_{j}\in K\,s\,\left(x_{i}\right)}D_{i\,j}\,x_{j}}{\sum_{x_{j}\in K\,s\,\left(x_{i}\right)}D_{i\,j}},$$

$${\hat{x}}_{i}=\frac{\sum_{x_{j}\in K\,s\,\left(x_{i}\right)}D_{i\,j}\,x_{j}}{\sum_{x_{j}\in K\,s\,\left(x_{i}\right)}D_{i\,j}},$$

where the *k* nearest neighbors of *x*_*i*_ are arranged in *Ks*(*x*_*i*_), and the Euclidean distance algorithm is used to find these neighbors. We design an undirected weight graph *G* = (*X*,*D*), where *D* ∈ ℝ*n*×*^n^* is weight matrix, *D*_*ji*_ = *D*_*ij*_≥0, and the element is measured as follows:


$$D_{i\,j}=\left\{ \begin{array}{cc} \exp\left(-\tau \,{||x_{i}-x_{j}||}^{2}\right), & x_{i}\,\mathrm{is}\,\mathrm{one}\,\mathrm{of}\,\mathrm{the}\,\mathrm{neighbors}\,\mathrm{of}\,x_{j}\\ 0 & \text{otherwise} \end{array}\right.,$$

where τ is a changeable parameter in Gaussian kernel function. If the distance between *x*_*i*_ and *x*_*j*_ is smaller, *D*_*ij*_ is higher, and *vice versa*. Therefore, the clustering problem is changed into a graph problem in this paper.

### Basic Formulation of MA-PCA

Since both SSPCA and SA-SSCCM methods require that the training data and test data meet the IID assumption, this paper reasonably combines the SSPCA method with a multi-model adaptation learning method (i.e., MA-PCA). This proposed method not only improves the robustness on noises/outliers but also solves the problems of insufficient label data and noisy data affecting the performance of the model and the different distribution of training data and test data. We therefore propose the following basic formula of MA-PCA:


(2) $$Q\,\left(W,{\text{v}}_{m}\,\left({\text{x}}_{j}\right),\gamma \right)=\min\Omega_{B}\,\left(W,{\text{v}}_{m}\,\left({\text{x}}_{j}\right)\right)+\beta \,\Omega_{M}\,\left(W,\gamma \right),$$

$$Q\,\left(W,{\text{v}}_{m}\,\left({\text{x}}_{j}\right),\gamma \right)=\min\Omega_{B}\,\left(W,{\text{v}}_{m}\,\left({\text{x}}_{j}\right)\right)+\beta \,\Omega_{M}\,\left(W,\gamma \right),$$

where Ω_*M*_(**W**,γ) is the multi-model adaptation term, and Ω_*B*_(**W**,*v*_*m*_(*x*_*j*_)) is used for reducing the negative impact of noises/outliers. We have the following function:


(3) $$\begin{array}{cc} & \Omega_{B}\,\left(W,{\text{v}}_{m}\,\left({\text{x}}_{j}\right)\right)\\ & =\min_{w,v_{m}\,\left(x_{j}\right)}\sum_{i=1}^{l}{||{\text{W}}^{T}\,{\text{x}}_{i}-{\text{y}}_{i}||}^{2}+\lambda_{s}\,\sum_{i=1}^{l}{||{\text{W}}^{T}\,{\hat{\text{x}}}_{i}-{\text{y}}_{i}||}^{2}\\ & +\sum_{m=1}^{M}\sum_{j=l+1}^{n}{\text{v}}_{m}^{2}\,\left({\text{x}}_{j}\right)\,{||{\text{W}}^{T}\,{\text{x}}_{j}-{\text{c}}_{m}||}^{2}\\ & +\lambda_{s}\,\sum_{m=1}^{M}\sum_{j=l+1}^{n}{\text{v}}_{m}^{2}\,\left({\text{x}}_{j}\right)\,{||{\text{W}}^{T}\,{\hat{\text{x}}}_{j}-{\text{c}}_{m}||}^{2}+\lambda \,{||{\text{W}}^{T}||}_{ℋ}^{2}\\ & +C\,\sum_{m=1}^{M}\sum_{j=l+1}^{n}\left({\text{v}}_{m}^{2}\,\left({\text{x}}_{j}\right)\,\ln{\text{v}}_{m}^{2}\,\left({\text{x}}_{j}\right)-v_{m}^{2}\,\left(x_{j}\right)\right)\\ & s.t\,.0\leq {\text{v}}_{m}\,\left({\text{x}}_{j}\right)\leq 1,m=1,\ldots ,M,j=l+1,\ldots ,n, \end{array}$$

$$\begin{array}{cc} & \Omega_{B}\,\left(W,{\text{v}}_{m}\,\left({\text{x}}_{j}\right)\right)\\ & =\min_{w,v_{m}\,\left(x_{j}\right)}\sum_{i=1}^{l}{||{\text{W}}^{T}\,{\text{x}}_{i}-{\text{y}}_{i}||}^{2}+\lambda_{s}\,\sum_{i=1}^{l}{||{\text{W}}^{T}\,{\hat{\text{x}}}_{i}-{\text{y}}_{i}||}^{2}\\ & +\sum_{m=1}^{M}\sum_{j=l+1}^{n}{\text{v}}_{m}^{2}\,\left({\text{x}}_{j}\right)\,{||{\text{W}}^{T}\,{\text{x}}_{j}-{\text{c}}_{m}||}^{2}\\ & +\lambda_{s}\,\sum_{m=1}^{M}\sum_{j=l+1}^{n}{\text{v}}_{m}^{2}\,\left({\text{x}}_{j}\right)\,{||{\text{W}}^{T}\,{\hat{\text{x}}}_{j}-{\text{c}}_{m}||}^{2}+\lambda \,{||{\text{W}}^{T}||}_{ℋ}^{2}\\ & +C\,\sum_{m=1}^{M}\sum_{j=l+1}^{n}\left({\text{v}}_{m}^{2}\,\left({\text{x}}_{j}\right)\,\ln{\text{v}}_{m}^{2}\,\left({\text{x}}_{j}\right)-v_{m}^{2}\,\left(x_{j}\right)\right)\\ & s.t\,.0\leq {\text{v}}_{m}\,\left({\text{x}}_{j}\right)\leq 1,m=1,\ldots ,M,j=l+1,\ldots ,n, \end{array}$$

where λ_*s*_,λ,*C* are balance parameters that can be adjusted to avoid overfitting during model training. The details about the other parameters are provided in Dan et al. (2021).

### The Multi-Model Adaptation Term in MA-PCA

In our domain adaptation learning, given is ${\left\{{\text{W}}_{i}^{s}\right\}}_{i=1}^{q}$ as a multiple-source model set, where *q* is source model number, and ${\text{W}}_{i}^{s}$ is the *i*-th source model. Each source model is obtained by learning the specified public dataset. This paper expects the classification results of the target model to be consistent with those of the source domain models. In other words, this paper will learn the target classifier *f*(*x*) = ^**W***T*^**x** in the whole sample space, regularize the weight parameters of different source domain models to control its complexity, and make each target instance be close to the source domain models. According to this criterion, it can be realized by introducing the multi-source domain model adaptation regularization function on the target domain. The formula is described as follows:


(4) $$\begin{array}{cc} & \Omega_{M}\,\left(W,\gamma \right)\\ & =\sum_{i=1}^{q}\gamma_{i}\,\int_{X}{||{\text{W}}^{T}\,\text{x}-{\text{W}}_{i}^{T}\,\text{x}||}^{2}\,d\text{x}+\eta \,{||\gamma ||}_{2}^{2}\\ & \overset{\Delta }{=}\sum_{i=1}^{q}\gamma_{i}\,\frac{1}{n}\,\sum_{j=1}^{n}{||{\text{W}}^{T}\,{\text{x}}_{j}-{\text{W}}_{i}^{s\,T}\,{\text{x}}_{j}||}_{F}^{2}+\eta \,{||\gamma ||}_{2}^{2}\\ & =\sum_{i=1}^{q}\gamma_{i}\,t\,r\,\left[{\left(\text{W}-{\text{W}}_{i}^{s}\right)}^{T}\,\text{S}\,\left(\text{W}-{\text{W}}_{i}^{s}\right)\right]+\eta \,{||\gamma ||}_{2}^{2}, \end{array}$$

$$\begin{array}{cc} & \Omega_{M}\,\left(W,\gamma \right)\\ & =\sum_{i=1}^{q}\gamma_{i}\,\int_{X}{||{\text{W}}^{T}\,\text{x}-{\text{W}}_{i}^{T}\,\text{x}||}^{2}\,d\text{x}+\eta \,{||\gamma ||}_{2}^{2}\\ & \overset{\Delta }{=}\sum_{i=1}^{q}\gamma_{i}\,\frac{1}{n}\,\sum_{j=1}^{n}{||{\text{W}}^{T}\,{\text{x}}_{j}-{\text{W}}_{i}^{s\,T}\,{\text{x}}_{j}||}_{F}^{2}+\eta \,{||\gamma ||}_{2}^{2}\\ & =\sum_{i=1}^{q}\gamma_{i}\,t\,r\,\left[{\left(\text{W}-{\text{W}}_{i}^{s}\right)}^{T}\,\text{S}\,\left(\text{W}-{\text{W}}_{i}^{s}\right)\right]+\eta \,{||\gamma ||}_{2}^{2}, \end{array}$$

where γ = ^[γ_1_,…,γ_*q*_]*T*^, and γ_*i*_ is a weight of the *i-th* source model. We constraint that the sum of γ_*i*_ is 1 (i.e., $\sum_{i=1}^{q}\gamma_{i}=1$). It is better to explore the contribution among the source models. The divergence matrix of the target domain is **S** = ^**XX***T*^. η ∈ *R*^+^ is a balance parameter to control the contribution of ${||\gamma ||}_{2}^{2}$. This parameter can be changed to avoid overfitting on multiple-source models. In addition, in the second equation in (4), we employ the sampling frequency as a weight to access the real distribution for the target domain.

**Remark 1**: The divergence matrix **S** is important for connecting the source classifiers and the target classifier. It will promote the learning of the target classification model to the real distribution direction of the target domain, thereby improving the generalization performance of adaptive learning, which is essentially different from other domain adaptation regularization terms based on DAL models (Bottou and Vapnik, 1992; Duan et al., 2012a). In order to better fit the model idea, this paper refers to formula (4) as the construction of the regularization term for divergence-constrained multi-model adaptation.

### Final Formulation

We expect better model adaptation performance for EEG-based emotion recognition by combining SSPCA with scatter-constrained multi-source classifier model. Therefore, a unified framework MA-PCA is obtained to learn **W**,**v**_*m*_(**x**_*j*_),γ by combining formulas (3) and (4). The optimization problem of MA-PCA can be described as follows:


$$Q\,\left(W,{\text{v}}_{m}\,\left({\text{x}}_{j}\right),\gamma \right)$$

$$Q\,\left(W,{\text{v}}_{m}\,\left({\text{x}}_{j}\right),\gamma \right)$$

$$\;=\min_{w,v_{m}\,\left(x_{j}\right)}\sum_{i=1}^{l}{||{\text{W}}^{T}\,{\text{x}}_{i}-{\text{y}}_{i}||}^{2}+\lambda_{s}\,\sum_{i=1}^{l}{||{\text{W}}^{T}\,{\hat{\text{x}}}_{i}-{\text{y}}_{i}||}^{2}$$

$$\;=\min_{w,v_{m}\,\left(x_{j}\right)}\sum_{i=1}^{l}{||{\text{W}}^{T}\,{\text{x}}_{i}-{\text{y}}_{i}||}^{2}+\lambda_{s}\,\sum_{i=1}^{l}{||{\text{W}}^{T}\,{\hat{\text{x}}}_{i}-{\text{y}}_{i}||}^{2}$$

$$\;+\sum_{m=1}^{M}\sum_{j=l+1}^{n}{\text{v}}_{m}^{2}\,\left(x_{j}\right)\,{||{\text{W}}^{T}\,{\text{x}}_{j}-{\text{c}}_{m}||}^{2}$$

$$\;+\sum_{m=1}^{M}\sum_{j=l+1}^{n}{\text{v}}_{m}^{2}\,\left(x_{j}\right)\,{||{\text{W}}^{T}\,{\text{x}}_{j}-{\text{c}}_{m}||}^{2}$$

$$\;+\lambda_{s}\,\sum_{m=1}^{M}\sum_{j=l+1}^{n}{\text{v}}_{m}^{2}\,\left({\text{x}}_{j}\right)\,{||{\text{W}}^{T}\,{\hat{\text{x}}}_{j}-{\text{c}}_{m}||}^{2}+\lambda \,{||{\text{W}}^{T}||}_{ℋ}^{2}$$

$$\;+\lambda_{s}\,\sum_{m=1}^{M}\sum_{j=l+1}^{n}{\text{v}}_{m}^{2}\,\left({\text{x}}_{j}\right)\,{||{\text{W}}^{T}\,{\hat{\text{x}}}_{j}-{\text{c}}_{m}||}^{2}+\lambda \,{||{\text{W}}^{T}||}_{ℋ}^{2}$$

$$\;+C\,\sum_{m=1}^{M}\sum_{j=l+1}^{n}\left({\text{v}}_{m}^{2}\,\left({\text{x}}_{j}\right)\,\ln{\text{v}}_{m}^{2}\,\left({\text{x}}_{j}\right)-{\text{v}}_{m}^{2}\,\left({\text{x}}_{j}\right)\right)$$

$$\;+C\,\sum_{m=1}^{M}\sum_{j=l+1}^{n}\left({\text{v}}_{m}^{2}\,\left({\text{x}}_{j}\right)\,\ln{\text{v}}_{m}^{2}\,\left({\text{x}}_{j}\right)-{\text{v}}_{m}^{2}\,\left({\text{x}}_{j}\right)\right)$$

(5) $$\;+\beta \,\left\{\begin{array}{c} \sum_{i=1}^{q}\gamma_{i}\,t\,r\,\left[{\left(\text{W}-{\text{W}}_{i}^{s}\right)}^{T}\,\text{S}\,\left(\text{W}-{\text{W}}_{i}^{s}\right)\right]\\ +\eta \,{||\gamma ||}_{2}^{2} \end{array}\right\},$$

$$\;+\beta \,\left\{\begin{array}{c} \sum_{i=1}^{q}\gamma_{i}\,t\,r\,\left[{\left(\text{W}-{\text{W}}_{i}^{s}\right)}^{T}\,\text{S}\,\left(\text{W}-{\text{W}}_{i}^{s}\right)\right]\\ +\eta \,{||\gamma ||}_{2}^{2} \end{array}\right\},$$

where γ = ^[γ_1_,γ_2_,…γ_*q*_]*T*^, γ ∈ ℝ*q*^×1^,γ*^T^*_**1***q*_ = 1, and *q* are the number of source domain models. When β = 0, MA-PCA degenerated to SSPCA. When β > 0, β is used as a balance parameter. When γ_*i*_ is constant 1, it indicates that there is only one single-source domain, and its distribution is the same as the target domain. At this time, MA-PCA approximates the SA-SSCCM method. When 0≤γ_*i*_ < 1, MA-PCA is a multi-model adaptation learning method based on the possibility clustering assumption, and the distribution of source domain and target domain can be identical or non-identical.

## Optimization

The objective function (5) is a non-convex function on (**W**,**v**_*m*_(**x**_*j*_),γ). In this paper, the strategy of alternating iterative optimization is adopted to realize the optimal solution of decision model **W**, membership model **v**_*m*_(**x**_*j*_), and contribution coefficient γ of the source models, respectively, and each iteration has an optimal solution.

### Update ***W*** as Given **v**_*m*_(**x**_*j*_) and γ

Firstly, fixing **v**_*m*_(**x**_*j*_) and γ to solve **W**: for ease of calculation, the following formula (5) is transformed into matrix form. **W** can be written as $\text{W}=\sum_{i=1}^{n}x_{i}\,\alpha_{i}$. Under the Representation Theorem, the formula (5) exists in reproducing kernel Hilbert space, and the kernel of *W* can be rewritten as follows: $W=\sum_{i=1}^{n}K\,\left(x_{i},x\right)\,\alpha_{i}$ (Belkin et al., 2006). Therefore, formula (5) is mapped into a finite dimensional space of the optimization α_*i*_ and can be reformulated as follows:


$$Q\,\left(\alpha \right)=\min_{\alpha }t\,r\,\left(\left(\alpha^{T}\,{\text{K}}_{l}\,{\text{K}}_{l}-Y\right)\,{\left(\alpha^{T}\,{\text{K}}_{l}\,{\text{K}}_{l}-Y\right)}^{T}\right)$$

Q⁢(α)

$$=\min_{\alpha }t\,r\,\left(\left(\alpha^{T}\,{\text{K}}_{l}\,{\text{K}}_{l}-Y\right)\,{\left(\alpha^{T}\,{\text{K}}_{l}\,{\text{K}}_{l}-Y\right)}^{T}\right)$$

$$\;+\lambda_{s}\,t\,r\,\left(\left(\alpha^{T}\,\bar{{\text{K}}_{l}}\,\bar{{\text{K}}_{l}}-\text{Y}\right)\,{\left(\alpha^{T}\,\bar{{\text{K}}_{l}}\,\bar{{\text{K}}_{l}}-Y\right)}^{T}\right)$$

$$\;+\lambda_{s}\,t\,r\,\left(\left(\alpha^{T}\,\bar{{\text{K}}_{l}}\,\bar{{\text{K}}_{l}}-\text{Y}\right)\,{\left(\alpha^{T}\,\bar{{\text{K}}_{l}}\,\bar{{\text{K}}_{l}}-Y\right)}^{T}\right)$$

$$\;+t\,r\,\left(\left(\alpha^{T}\,{\text{K}}_{u}\,{\text{K}}_{u}\,\text{J}-\text{L}\right)\,\hat{\text{V}}\,{\left(\alpha^{T}\,{\text{K}}_{u}\,{\text{K}}_{u}\,\text{J}-\text{L}\right)}^{T}\right)$$

$$\;+t\,r\,\left(\left(\alpha^{T}\,{\text{K}}_{u}\,{\text{K}}_{u}\,\text{J}-\text{L}\right)\,\hat{\text{V}}\,{\left(\alpha^{T}\,{\text{K}}_{u}\,{\text{K}}_{u}\,\text{J}-\text{L}\right)}^{T}\right)$$

$$\;+\lambda_{s}\,t\,r\,\left(\left(\alpha^{T}\,\bar{{\text{K}}_{u}}\,\bar{{\text{K}}_{u}}\,\text{J}-\text{L}\right)\,\hat{\text{V}}\,{\left(\alpha^{T}\,\bar{{\text{K}}_{u}}\,\bar{{\text{K}}_{u}}\,\text{J}-\text{L}\right)}^{T}\right)$$

$$\;+\lambda_{s}\,t\,r\,\left(\left(\alpha^{T}\,\bar{{\text{K}}_{u}}\,\bar{{\text{K}}_{u}}\,\text{J}-\text{L}\right)\,\hat{\text{V}}\,{\left(\alpha^{T}\,\bar{{\text{K}}_{u}}\,\bar{{\text{K}}_{u}}\,\text{J}-\text{L}\right)}^{T}\right)$$

$$\;+\lambda \,t\,r\,\left(\alpha^{T}\,\text{KK}\,\alpha \right)+\beta \,\sum_{i=1}^{a}\gamma_{i}\,\left({\text{W}}_{I}^{T}-\alpha^{T}\,\text{K}\right)\,\text{K}$$

$$\;+\lambda \,t\,r\,\left(\alpha^{T}\,\text{KK}\,\alpha \right)+\beta \,\sum_{i=1}^{a}\gamma_{i}\,\left({\text{W}}_{I}^{T}-\alpha^{T}\,\text{K}\right)\,\text{K}$$

(6) $$\;\cdot \text{K}\,\left({\text{W}}_{i}-\text{K}\,\alpha \right),$$

$$\;\cdot \text{K}\,\left({\text{W}}_{i}-\text{K}\,\alpha \right),$$

where the details about $J,L,\hat{\text{V}}$ can be found in Dan et al. (2021).

By solving the derivation of (6) w.r.t. α and letting it be equal to 0 (i.e., ∂⁡*Q*(α)/∂⁡α = 0), we obtain the following:


(7) $$\alpha ={\text{H}}^{-1}\,\text{B},$$

$$\alpha ={\text{H}}^{-1}\,\text{B},$$

where


$$\text{H}={\text{K}}_{l}\,{\text{K}}_{l}\,{\text{K}}_{l}\,{\text{K}}_{l}+\lambda \,\bar{{\text{K}}_{l}}\,\bar{{\text{K}}_{l}}\,\bar{{\text{K}}_{l}}\,\bar{{\text{K}}_{l}}$$

$$\;+{\text{K}}_{u}\,{\text{K}}_{u}\,\text{J}\,\hat{\text{V}}\,{\text{J}}^{T}\,{\text{K}}_{u}\,{\text{K}}_{u}$$

$$\;+\lambda_{s}\,\bar{{\text{K}}_{u}}\,\bar{{\text{K}}_{u}}\,\text{J}\,\hat{\text{V}}\,{\text{J}}^{T}\,\bar{{\text{K}}_{u}}\,\bar{{\text{K}}_{u}}+\lambda \,\text{KK}+\beta \,\sum_{i=1}^{q}\gamma_{i}\,\text{KKKK},$$

and


$$\text{B}={\text{K}}_{l}\,{\text{K}}_{l}\,{\text{Y}}^{T}+\lambda_{s}\,\bar{{\text{K}}_{l}}\,\bar{{\text{K}}_{l}}\,Y^{T}+{\text{K}}_{u}\,{\text{K}}_{u}\,\text{J}\,\hat{\text{V}}\,\text{L}$$

$$\;+\lambda_{s}\,\bar{{\text{K}}_{u}}\,\bar{{\text{K}}_{u}}\,\text{J}\,\hat{\text{V}}\,{\text{L}}^{T}+\beta \,\sum_{i=1}^{q}\gamma_{i},{\text{KKKW}}_{i}^{s},$$

Finally, the solution of **W** is **K**α.

### Update **v**_*m*_(**x**_*j*_) as Given **W** and γ

Then, we fix **W** and γ to solve **v**_*m*_(**x**_*j*_). The optimal problem of the objective function in (5) can be rewritten as follows:


(8) $$\begin{array}{cc} & Q\,\left({\text{v}}_{m}\,\left({\text{x}}_{j}\right)\right)\\ & =\min_{v_{m}\,\left(x_{j}\right)}\sum_{m=1}^{M}\sum_{j=l+1}^{n}v_{m}\,{\left(x_{j}\right)}^{2}\,{||{\text{W}}^{T}\,{\text{x}}_{j}-{\text{c}}_{m}||}^{2}\\ & +\lambda_{s}\,\sum_{m=1}^{M}\sum_{j=l+1}^{n}v_{k}\,{\left(x_{j}\right)}^{2}\,{||{\text{W}}^{T}\,{\hat{\text{x}}}_{j}-{\text{c}}_{m}||}^{2}+\lambda \,{||{\text{W}}^{T}||}_{ℋ}^{2}\\ & +C\,\sum_{m=1}^{M}\sum_{j=l+1}^{n}\left({\text{v}}_{m}\,{\left({\text{x}}_{j}\right)}^{2}\,\ln{\text{v}}_{m}\,{\left({\text{x}}_{j}\right)}^{2}-{\text{v}}_{m}\,{\left({\text{x}}_{j}\right)}^{2}\right), \end{array}$$

$$\begin{array}{cc} & Q\,\left({\text{v}}_{m}\,\left({\text{x}}_{j}\right)\right)\\ & =\min_{v_{m}\,\left(x_{j}\right)}\sum_{m=1}^{M}\sum_{j=l+1}^{n}v_{m}\,{\left(x_{j}\right)}^{2}\,{||{\text{W}}^{T}\,{\text{x}}_{j}-{\text{c}}_{m}||}^{2}\\ & +\lambda_{s}\,\sum_{m=1}^{M}\sum_{j=l+1}^{n}v_{k}\,{\left(x_{j}\right)}^{2}\,{||{\text{W}}^{T}\,{\hat{\text{x}}}_{j}-{\text{c}}_{m}||}^{2}+\lambda \,{||{\text{W}}^{T}||}_{ℋ}^{2}\\ & +C\,\sum_{m=1}^{M}\sum_{j=l+1}^{n}\left({\text{v}}_{m}\,{\left({\text{x}}_{j}\right)}^{2}\,\ln{\text{v}}_{m}\,{\left({\text{x}}_{j}\right)}^{2}-{\text{v}}_{m}\,{\left({\text{x}}_{j}\right)}^{2}\right), \end{array}$$

By solving the derivation of Eq. (5) w.r.t. **v**_*m*_(**x**_*j*_) and letting it be equal to zero (i.e., ∂⁡*Q*(**v**_*m*_(**x**_*j*_))/∂⁡*v*_*m*_(*x*_*j*_) = 0), then


$$\partial Q\,\left({\text{v}}_{m}\,\left({\text{x}}_{j}\right)\right)\,/\,\partial {\text{v}}_{m}\,\left({\text{x}}_{j}\right)=2\,{\text{v}}_{m}\,\left({\text{x}}_{j}\right)\,{||{\text{W}}^{T}\,{\text{x}}_{j}-c_{m}||}^{2}$$

$$\;+2\,\lambda_{s}\,{\text{v}}_{m}\,\left({\text{x}}_{j}\right)\,{||{\text{W}}^{T}\,{\hat{x}}_{j}-c_{m}||}^{2}$$

$$\;+C\,\left[2\,{\text{v}}_{m}\,\left({\text{x}}_{j}\right)\,\log{\text{v}}_{m}\,{\left({\text{x}}_{j}\right)}^{2}\right]=0,$$

we can get:


(9) $${\text{v}}_{m}\,\left({\text{x}}_{j}\right)=\exp\left(\frac{-\left({||{\text{W}}^{T}\,{\text{x}}_{j}-c_{m}||}^{2}+{||{\text{W}}^{T}\,{\hat{x}}_{j}-c_{m}||}^{2}\right)}{2\,C}\right),$$

Since **x**_*j*_ means any one instance, the general presentation of **v**_*m*_(**x**_*j*_) is as follows:


(10) $${\text{v}}_{m}\,\left(x\right)=\exp\left(\frac{-\left({||{\text{W}}^{T}\,\text{x}-{\text{c}}_{m}||}^{2}+{||{\text{W}}^{T}\,\hat{\text{x}}-{\text{c}}_{m}||}^{2}\right)}{2\,C}\right),$$

$${\text{v}}_{m}\,\left(x\right)=\exp\left(\frac{-\left({||{\text{W}}^{T}\,\text{x}-{\text{c}}_{m}||}^{2}+{||{\text{W}}^{T}\,\hat{\text{x}}-{\text{c}}_{m}||}^{2}\right)}{2\,C}\right),$$

### Update γ by Fixing **W** and **v**_*m*_(**x**_*j*_)

We define ${\text{A}}_{i}=tr\left[{\left(\text{W}-{\text{W}}_{i}^{s}\right)}^{T}\text{S}\left(\text{W}-{\text{W}}_{i}^{s}\right)\right]\left(i=1,\ldots ,q\right)$ which is corresponding to the adaptation of the *i*-th source model to the target domain. Given **W** and **v**_*m*_(**x**_*j*_), the objective function (5) can be rewritten in (11) as follows:


$$\min_{\gamma }\gamma^{T}\,\text{A}+\eta \,{||\gamma ||}_{2}^{2}$$

$$\min_{\gamma }\gamma^{T}\,\text{A}+\eta \,{||\gamma ||}_{2}^{2}$$

(11) $$s.t.\gamma^{T}1</cps:bf{>}_{q}=1,0\leq \gamma \leq 1,$$

$$s.t.\gamma^{T}1</cps:bf{>}_{q}=1,0\leq \gamma \leq 1,$$

where **A** = ^(**A**_1_,…,**A**_*q*_)*T*^. The optimal estimation of γ becomes the optimal weight division problem of multiple model adaptation learning with scatter constraints. Theoretically, if η = 0, the optimal **γ_*i*_** will be 1; otherwise, if **A**_*i*_ = *min*_*j* = 1,…,*q*_⁡**A**_*j*_, γ_*i*_ is 0; if η→ + ∞, the optimal γ_*i*_ will tend to be the same weight 1/*q*. Thus, the following theorem is obtained:

**Theorem 1** (Karasuyama and Mamitsuka, 2013). The following equation is the optimal solution of (11):


$$\gamma_{i}=\left\{ \begin{array}{cc} \frac{\rho -{\text{A}}_{i}}{}\,\eta ,i=1,2,\ldots ,\zeta & \\ 0, & i=\zeta +1,\ldots ,q \end{array}\right.,$$

where $\rho =\left(\eta +\sum_{i=1}^{q}{\text{A}}_{i}\right)\,/\,\zeta$, ζ = |{i|ρ−**A**_*i*_ > 0,*i* = 1,2,…,*q*}|.

Theorem 1 presents that there are ζnon-zero entries in the optimal γ. According to the target domain, this optimization can select source domains automatically with a different γ. If γis bigger, there is a higher similarity between the source domain and the target domain. Since the optimal γcould be calculated *via* the optimal ζ, Karasuyama and Mamitsuka, 2013) presented a special method to obtain the optimal ζ. This algorithm effectiveness can be proved under the given right amount of source domains.

Next, the algorithm adopts a coordinate descent strategy to solve (11), which is close to the method in Geng et al. (2012). In each iteration cycle, when other entries are fixed, we select two items to update, and γ*^T^*_1*q*_ = 1 must be satisfied at the end of each iteration. Suppose that in an iteration cycle the *i*-th and *j*-th entries are selected, the following iterative formula can therefore be obtained:


(12) $$\left\{ \begin{array}{c} \left(\text{I}\right)\,\gamma_{i}^{*}=0,\gamma_{j}^{*}=\gamma_{i}+\gamma_{j},i\,f\,2\,\eta \,\left(\gamma_{i}+\gamma_{j}\right)+\left({\text{A}}_{j}-{\text{A}}_{i}\right)\leq 0\\ \left(\text{II}\right)\,\gamma_{j}^{*}=0,\gamma_{i}^{*}=\gamma_{i}+\gamma_{j},i\,f\,2\,\eta \,\left(\gamma_{i}+\gamma_{j}\right)+\left({\text{A}}_{i}-{\text{A}}_{j}\right)\leq 0\\ \left(\text{III}\right)\,o\,r\,\gamma_{i}^{*}=1/2\,\left(\gamma_{i}+\gamma_{j}\right)+1/4\,\eta \,\left({\text{A}}_{j}-{\text{A}}_{i}\right),\\ \gamma_{j}^{*}=\gamma_{i}+\gamma_{j}-\gamma_{i}^{*}. \end{array}\right.,$$

$$\left\{ \begin{array}{c} \left(\text{I}\right)\,\gamma_{i}^{*}=0,\gamma_{j}^{*}=\gamma_{i}+\gamma_{j},i\,f\,2\,\eta \,\left(\gamma_{i}+\gamma_{j}\right)+\left({\text{A}}_{j}-{\text{A}}_{i}\right)\leq 0\\ \left(\text{II}\right)\,\gamma_{j}^{*}=0,\gamma_{i}^{*}=\gamma_{i}+\gamma_{j},i\,f\,2\,\eta \,\left(\gamma_{i}+\gamma_{j}\right)+\left({\text{A}}_{i}-{\text{A}}_{j}\right)\leq 0\\ \left(\text{III}\right)\,o\,r\,\gamma_{i}^{*}=1/2\,\left(\gamma_{i}+\gamma_{j}\right)+1/4\,\eta \,\left({\text{A}}_{j}-{\text{A}}_{i}\right),\\ \gamma_{j}^{*}=\gamma_{i}+\gamma_{j}-\gamma_{i}^{*}. \end{array}\right.,$$

We iteratively traverse all paired entries in γ and optimize any two entries in γ by (12) until the optimization function (5) converges. Intuitively, the updating criteria in (12) tends to be that the larger the value to γ_*i*_, the smaller the **A**_*i*_. Since **A**_*i*_ measures the distribution distance between the *i*-th source model and the target domain, the smaller the **A**_*i*_, the higher the correlation between the *i*-th source domain and the target domain.

**Remark 1**: After obtaining the optimal solution of *W* and *V*, the label matrix of the samples in the target domain can also be obtained, and the influence of noise has been effectively suppressed. The label and the membership of out of sample can also be calculated by *W* and *V*. Finally, the performance of *W* relies on the learned graph *G = XD* and the multiple-source domain adaptation model.

### Convergence Analysis

Since the objective function (5) is a multi-objective optimization function, it is difficult to guarantee the global optimal solution. It is worthy to note that the algorithm in this paper adopts an alternate iterative strategy for optimizing. Since the objective function of a single optimization variable [i.e., equations (6), (8), and (11)] is convex and in closed form, the iterative optimal solution can be obtained. Therefore, this work only deduces the asymptotic convergence of the algorithm based on the iterative target value of the objective function. The derivation process is shown in (13) as follows:


(13) $$\begin{array}{cc} & Q\,\left(W^{\text{itr}},v^{i\,t\,r},\gamma^{i\,t\,r}\right)\geq \\ & Q\,\left(W^{i\,t\,r+1},v^{i\,t\,r},\gamma^{i\,t\,r}\right)\geq \\ & Q\,\left(W^{i\,t\,r+1},v^{i\,t\,r+1},\gamma^{i\,t\,r}\right)\geq \\ & Q\,\left(W^{i\,t\,r+1},v^{i\,t\,r+1},\gamma^{i\,t\,r+1}\right)>\varepsilon >0, \end{array}$$

$$\begin{array}{cc} & Q\,\left(W^{\text{itr}},v^{i\,t\,r},\gamma^{i\,t\,r}\right)\geq \\ & Q\,\left(W^{i\,t\,r+1},v^{i\,t\,r},\gamma^{i\,t\,r}\right)\geq \\ & Q\,\left(W^{i\,t\,r+1},v^{i\,t\,r+1},\gamma^{i\,t\,r}\right)\geq \\ & Q\,\left(W^{i\,t\,r+1},v^{i\,t\,r+1},\gamma^{i\,t\,r+1}\right)>\varepsilon >0, \end{array}$$

where ^Witr^,^v*itr*^,^γ*itr*^ are the optimal solutions at the *itr*-th iteration. ε is a very small constant. The objective function will converge to a local optimum. The derivation process proves that the iterative target value of the algorithm shows a downward trend. When the value drops to a certain threshold (at least greater than ε), we stop the iterative. Finally, the objective function will converge to the local target value of each single optimization variable.

## Algorithm Description

The optimization of MA-PCA adopts an alternating iteration strategy. The most semi-supervised learning methods are often optimized by iterative learning. In addition, the membership value of initialized unlabeled instances can be obtained by any of the following methods: some fuzzy clustering method, randomization strategy, or all initialized to zero. Therefore, the learning of MA-PCA method starts with labeled instances to initialize the decision model *W*. When the objective function converges, the iteration terminates. The algorithm in this paper specifically adopts a window-based stopping criterion to better control the algorithm convergence: given a window size ℏ, computing ς = |*Max*Θ_*itr*_−*Min*Θ_*itr*_|/MaxΘ_itr_ at the *itr*-th iteration (Θ_*itr*_ = {*Obj*_*itr*−ℏ + 1_,…,*Obj*_*itr*_}means Θ_*itr*_is composited by the historical target value in this window. When ς < ε, the iteration terminates. The details of this algorithm are shown in Table 1.

**TABLE 1:** Algorithm description of MA-PCA.

**Input**: the target domain with data X_*l*_ and its labels Y_*l*_, unlabeled data X_*u*_, regular term parameter λ,λ_*s*_,*C*,η,β. There are *q* source classifier models ${\left\{{\text{W}}_{i}^{s}\right\}}_{i=1}^{q}$, iteration termination threshold ε, and maximum number of iterations *N.*
**Output**: the target classifier model W, the label membership function v, and the contribution coefficient γ of multi-source model.
**Procedure**:
1. Initialize the label memberships of unlabeled data, $\gamma_{i}^{0}=1/q\left(\text{i}=1,\ldots ,q\right)$;
2. Obtain the initial ^W0^ by Eq. (7);
3. Obtain the initial ^v0^ by Eq. (10);
4. Calculate the *Q*(^W0^,*v*^0^,γ^0^) of objective function
**for** *itr* = 1 **to** *N* **do**
{
5.1 Fix the current ^v*itr*^ and ^γ*itr*^ for updating ^W*itr*^ to ^W*itr* + 1^ by Eq. (7)
5.2 Fix the current ^W*itr*^ and ^γ*itr*^ for updating ^v*itr*^ to ^v*itr* + 1^ by Eq. (10)
5.3 Fix the current ^W*itr*^ and ^v*itr*^ for updating ^γ*itr*^ to ^γ*itr* + 1^ by Eq. (12)
**Until** *itr* > *N*orς < ε, return the optimal W, v, and γ
}

## Experiment

In this part, we comprehensively compare the proposed method with several state-of-the-arts on two widely used benchmark databases, including SEED (Zheng and Lu, 2015) and DEAP (Koelstra et al., 2012), for EEG-based emotion recognition (Mansour et al., 2009).

### Datasets

According to Lan et al. (2018) and Zhong et al. (2020), there exist certain significant differences between SEED and DEAP since they can be generated by different subjects, sessions, EEG devices, experimental schemes, emotional stimuli, etc., Detailed information about these two datasets can be viewed in Lan et al. (2018). In the following experiments, we adopt differential entropy (Lan et al., 2018; Zhong et al., 2020) as the data feature in emotion recognition, which has also been widely used in the preceding literatures (Shi et al., 2015; Zheng et al., 2015, 2016; Chai et al., 2016, 2017; Lan et al., 2018; Zhong et al., 2020) for domain adaptation emotion recognition.

### Baseline Setting

We will systematically compare our method with such state-of-the-arts as SSPCA (Dan et al., 2021), a baseline without domain adaptation, FastDAM (Duan et al., 2012b), Multi-KT (Tommasi et al., 2014) with *l*_*2*_-norm constraint on *p*, A-SVM (Yang et al., 2007), and DSM (Duan et al., 2012a). Since existing deep domain adaptation frameworks have achieved many inspiring results on emotion recognition as well as visual recognition, we also additionally present comparisons with several deep (CNN-based) domain adaptation methods with deep features: DAN (Long et al., 2015) and Reverse Grad (Ganin and Lempitsky, 2015).

It was noted that, in the DA schema, automatic parameter tuning is not possible for source classifiers using cross-validation due to the reason that training and test data are from different data distributions. Therefore, all methods evaluated in this paper on the dataset are empirically searched for the parameter space to optimize parameter settings to obtain the best results for each method. The parameters of all methods are adjusted to obtain the best results, except for the specially specified parameters.

For the method SSPCA without domain adaptation, this experiment will fuse the decision values of all classifiers obtained from the independent training of labeled samples in each source domain and target domain. DSM, Multi-KT, A-SVM, and FastDAM are domain adaptation methods. For A-SVM, this experiment also fuses the decision values of all basic classifiers, and each classifier is learned from a labeled sample in a source domain.^1^

There are some hyper-parameters in the objective function (5) that need to be determined. First of all, this experiment sets the most important parameters (e.g., γ_*i*_) as optimization variables in the iterative optimization process, and only a few crucial parameters that are proposed in MA-PCA need to be pre-defined (e.g., λ,λ_*s*_,*C*,η,β), and considering that parameter determination is an open problem in the field of machine learning, we have determined parameters empirically in a past work. Since the exponent of γ_*i*_ plays the role of avoiding trivial solutions in the process of optimizing γ_*i*_, as proved in Hou et al. (2017), the larger the exponent of γ_*i*_ is, the closer all weight values are to be same. In order to reflect the differences among different source domains, this experiment will set the index of γ_*i*_ to be 2 by experience. The validity of this decision will be verified in the experimental results in the following section. The hyperparameter λ,λ_*s*_,*C*,η,β is adjusted within the range of {10^−4^,10^−3^,…,10^3^,10^4^}. Finally, we search the nearest neighbor number *k* from the set {35,10,15,17} to construct the nearest neighbor graph in MA-PCA (also SSPCA) and obtain the first-ranked recognition accuracy from the optimal parameter configuration.

For non-linear learning methods MA-PCA, FastDAM, and multi-KT, Gaussian kernel *K*_*i*,*j*_ = *exp*⁡(−σ^||*x*_*i*_−x_*j*_||2^) is default kernel function, σ = 1/*d*, and *d* is the feature dimension. In FastDAM, $\gamma_{i}=\frac{\exp\left(-\delta \,D\,i\,s\,t\,\left(X_{i}^{s},X\right)\right)}{\sum_{i}\exp\left(-\delta \,D\,i\,s\,t\,\left(X_{i}^{s},X\right)\right)}$ (*i* = 1,…,*S*) is the weight value of each source domain, δ = 100. For the benchmark method SSPCA, the target domain samples are directly mapped to the source domain without any domain adaptation, and the decision values of all classifiers obtained from the independent training of labeled samples from each source domain and target domain are equally fused.

### Emotion Recognition Within Dataset

Note that different subjects even from the same dataset still have different EEG feature distributions due to individual characteristics. We therefore practice the so-called leave-one-out cross-validation strategy conducted also in Lan et al. (2018) to evaluate the emotion recognition performance of MA-PCA—that is, one subject remained to be the target domain, and the others from the dataset are constructed as multiple sources. In this multi-source scenario, we follow the same setting as Tao et al. (2021) to evaluate our method compared with other state-of-the-arts on SEED and DEAP, respectively.

#### Performance Comparison

**FIGURE 1:**
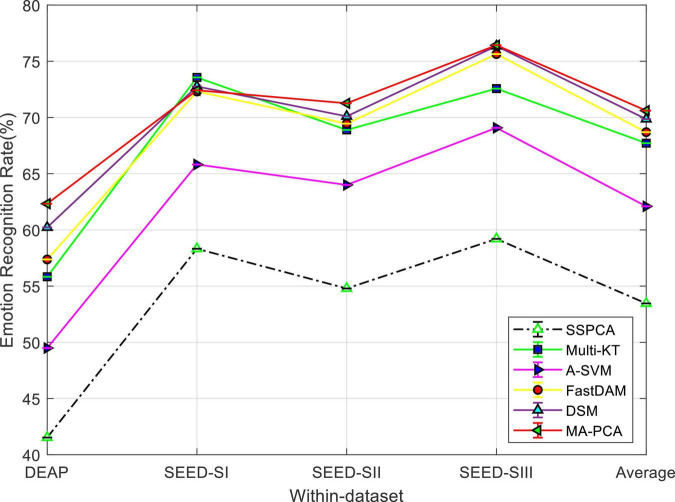
Domain adaptation emotion recognition on within dataset. SI, session I; SII, session II; SIII, session III.

The emotion recognition performance of MA-PCA and the rest of the comparison methods within the DEAP and SEED datasets are visualized in Figure 1. It can be seen from the bar plot that the recognition performance of all DA methods is better than that of SSPCA, and MA-PCA achieves the best performance (about 21% performance improvement over SSPCA), followed by DSM on DEAP dataset. Besides this, those multi-source adaptation methods, including our method, unsurprisingly achieved more accuracy gains than the no-adaptation method SSPCA on SEED. We can observe that our method MA-PCA demonstrates the best performance on SEED by upgrading the average accuracy. An interesting observation is that all methods work better on SEED than on DEAP, which has also been reported in Lan et al. (2018) and Tao et al. (2021). The reason for this phenomenon might be that the larger distribution discrepancy between different subjects from DEAP prevented boosting performance in these methods (Mansour et al., 2009; Lan et al., 2018).

Finally, MA-PCA achieved almost the best performance on both datasets. A possible explanation is that the distribution discrepancy may exist in the same dataset (i.e., DEAP or SEED), and MA-PCA can learn a more robust target classifier for domain adaptation by discriminatively selecting a set of prelearned base classifiers in the non-IID scenario of multi-subject adaptation.

#### Multi-Kernel Learning

As well known, the choice of kernel is a challenging issue in the kernel learning method. Recently, multiple kernel learning (MKL) has been effectively proposed for conquering this choice issue that existed in single kernel learning methods. Consequently, we also evaluate the performance boost in our method by using MKL (called MKMA-PCA for short) for each source domain. To this end, the first step is to construct a new space spanned by multiple kernel mapping features. We firstly denote by ${\left\{\phi_{a}\right\}}_{a=1}^{℧}$ an empirical kernel function set, which, respectively, projected *X*_*a*_ into ℧ different spaces. Then, an orthogonally integrated space can be constructed by concatenating these ℧ spaces. We denote the mapping features in this final space by $\tilde{\phi }\,\left(x_{i}\right)={\left[\phi_{1}\,{\left(x_{i}\right)}^{T},\phi_{2}\,{\left(x_{i}\right)}^{T},\ldots ,\phi_{℧}\,{\left(x_{i}\right)}^{T}\right]}^{T}\in {\mathbb{R}}^{℧\,n_{a}}$, where *x*_*i*_ ∈ *X*_*a*_. Correspondingly, the kernel matrix in this final space can be easily deduced as $K_{n\,e\,w}=\left[{\tilde{K}}_{1};{\tilde{K}}_{2};\ldots ;{\tilde{K}}_{℧}\right]$, where ${\tilde{K}}_{i}$ is the *i*-th kernel matrix from the ℧ feature spaces. Aiming to exploit the multiple kernel spaces, we therefore employ four kernel mapping functions, including the Gaussian kernel used above. The other additionally employed kernels are inverse square distance kernel function, Laplacian kernel function, and inverse distance kernel function, respectively, denoted as K_*ij*_ = 1/(1 + σ^||*x*_*i*_−x_*j*_||2^), ${\text{K}}_{i\,j}=\exp\left(-\sqrt{\sigma }\,||x_{i}-{\text{x}}_{j}||\right)$, and ${\text{K}}_{i\,j}=1/\left(1+\sqrt{\sigma }\,||x_{i}-{\text{x}}_{j}||\right)$.

**FIGURE 2:**
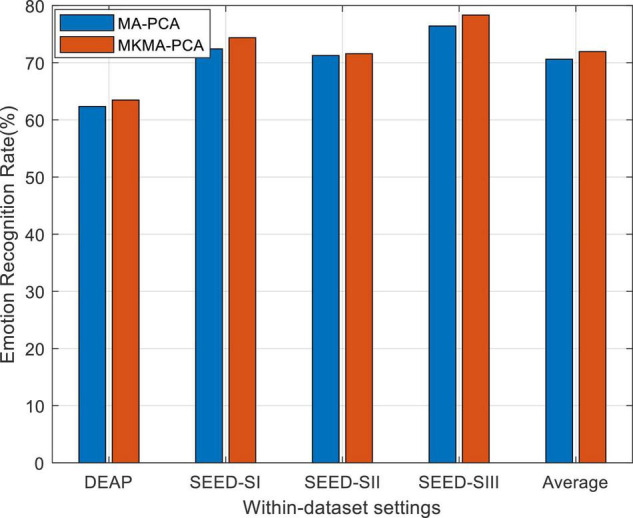
Emotion recognition on within-dataset with multiple kernel learning. SI, session I; SII, session II; SIII, session III (similarly hereinafter).

The observation from Figure 2, in which MKMA-PCA significantly outperforms MA-PCA, justifies that our MA-PCA with MKL can further boost the recognition performance on DEAP and SEED. This also proves the importance of kernel choice in those kernel-based learning models.

### Emotion Recognition Cross-Dataset

It is more challenging on emotion recognition when across datasets, with the differences in acquisition pathways, subjects’ characteristics, and behaviors. The previous experiments show the performance comparison of MA-PCA with other DA methods within the dataset (i.e., across subjects). This subsection further evaluates the robust effectiveness of MA-PCA when adapting across datasets. In this experimental scenario, multiple different protocols were constructed using different EEG devices and emotional stimuli by sampling the training and testing datasets separately. Therefore, six experimental settings, namely, DEAP → session I, DEAP → session II, DEAP → session III, session I → DEAP, session II → DEAP, and session III → DEAP, were set up to demonstrate that MA-PCA has robust effectiveness on emotion recognition with cross-dataset. For simplicity of expression, session I, session II, and session III in SEED are coded as SI, SII, and SIII, respectively, [for detailed experimental setup information, see the literature Tao et al. (2021)].

#### Performance Comparison

**FIGURE 3:**
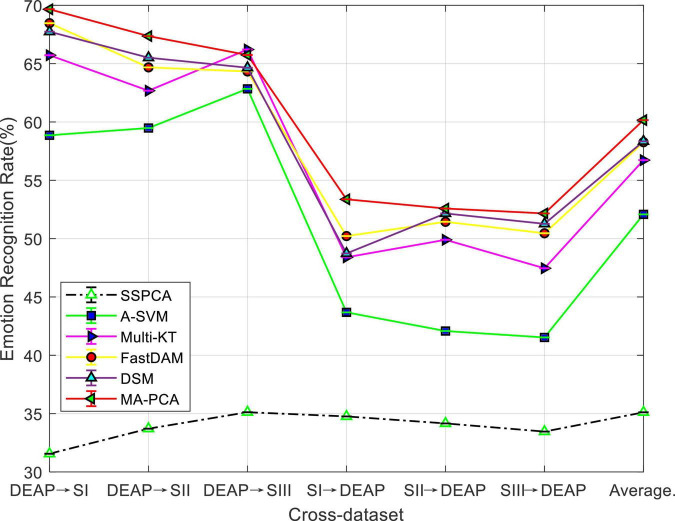
Domain adaptation emotion recognition on cross-dataset.

We aim to evaluate the performance of our method MA-PCA using the emotion recognition results on cross-dataset from DEAP and SEED. The experimental results are, respectively, plotted in Figure 3, which shows the average results for six possible combinations.

It can be seen from the bars in Figure 3 that the no-adaptation method SSPCA has the worst performance than others in all cases, which witnesses the existence of distribution discrepancy between DEAP and one of the sessions from SEED. In this context, the importance of domain adaptation (DA) will be indispensable. This is justified by the observation in Figure 3 that DA may reduce technical differences in cross-dataset applications, and our MA-PCA consistently outperformed other DA methods in most cases of the cross-dataset settings. A possible reason for this may be that the clustering hypothesis with fuzzy entropy in our MA-PCA could weaken the impact of noise from these datasets. A clear phenomenon can be observed in Figure 3—due to the large distribution difference between the different datasets, the average recognition accuracy of all methods is correspondingly lower than the results obtained in Figure 1 within the dataset.

#### Emotion Recognition With Multi-Source Prior

**FIGURE 4:**
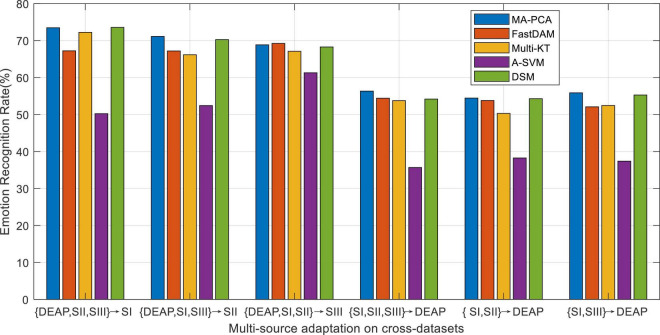
Emotion recognition with multi-source adaptation settings.

As reported in preceding works about domain adaptation learning, multiple-source domains can improve the adaptation performance to some extent by integrating multiple prior knowledge. Nevertheless, in concrete applications, multi-source adaptation also incurs another challenge, i.e., source scalability issue, since multi-source learning could lead to the so-called negative transfer problem. In this scenario, how to discriminately exploit multiple sources becomes a challenge worthy to be addressed in multi-source adaptation learning frameworks. To this end, we will explore in this part the different reliabilities of the prior sources in the emotion recognition task (Tao et al., 2019). We evaluate the performance of all baseline domain adaptation methods with multiple prior sources on the designed cross-dataset settings. The average accuracies of all methods are plotted in Figure 4, where A-SVM employs the average prior model.

When there exists a very large distribution discrepancy between different domain datasets, it is hard for A-SVM to eliminate the inter-domain distribution bias. Therefore, the results in Figure 4 shows that A-SVM is inferior compared with the other multi-source adaptation methods in most settings. A-SVM even has a downgraded performance tendency with the increase of source domains in some scenarios, which indicates the existence of “negative transfer” phenomenon in A-SVM. Another interesting observation from Figure 4 is that all DA methods, except A-SVM, achieve more improvement by leveraging multiple-source knowledge than that by bridging only one source (i.e., cross-subject settings) when the number of source domains increases. This proves that it is beneficial to leverage multiple sources for boosting the recognition performance. Moreover, MA-PCA and DSM conquer others by touching on the top performance due to their designed weights for discriminately screening the optimal sources. Our method MA-PCA obtains more gains over DSM in some scenarios. This may be attributed to the adopted strategy in MA-PCA, which can efficiently select the most relevant source domains through optimal weighted multi-source adaptive regularization.

#### Adaptive Emotion Recognition With Deeply Extracted Features

In this subsection, we will particularly evaluate our method MA-PCA with deeply extracted features by comparing it with several recently proposed deep adaptation models on cross-dataset emotion recognition using multi-source settings.

In practical tasks, our method MA-PCA can be trained on the deeply transformed features of all domains, which follows the same setup with that in Zhu et al. (2017) and Zhou et al. (2018). Concretely, some pre-trained deep models (e.g., VGG16 and DAN, etc.) are first fine-tuned using the source domain, then the deep features can be extracted from EEG signals in both source and target domains with this CNN model, and finally the recognition model would be trained on these extracted features. In the context of our experiments, we denote our methods with VGG16 (respectively DAN) model as MA-PCA + VGG16 (MA-PCA + DAN respectively). As for DAN and ReverseGrad, we use their released source codes to fine-tune the pre-trained models by, respectively, using the pre-tuned parameters in their works (Ganin and Lempitsky, 2015; Long et al., 2015). Note that these deep adaptation methods typically aim to learn domain-invariant representations. Differently from the deep adaptation frameworks, our proposed method explores to learn a domain-invariant recognition model with strong generalization ability from the source domain to the target one.

**FIGURE 5:**
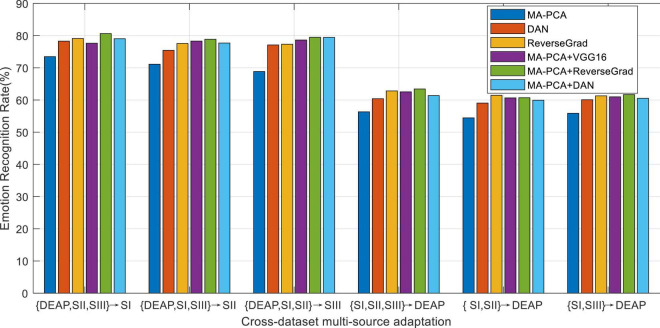
Adaptive emotion recognition using deeply extracted features.

We plot the mean results of all methods in Figure 5, from which we can observe that our deep adaptation method MA-PCA + VGG16 and MA-PCA + DAN significantly outperform MA-PCA. This indicates the advantage of deep features due to its robust feature representation. Furthermore, MA-PCA + DAN also obtains comparable recognition performance with respect to other deep adaptation methods. This may be attributed to the classification-level constraint in MA-PCA, where most of the source discriminative structures are expected to be preserved by the guidance of target classification. In some cases, shown in Figure 5, MA-PCA + VGG16 or MA-PCA + DAN even achieves the top-one performance compared with other deep adaptation frameworks. This phenomenon shows that the proposed MA-PCA can become an effective surrogate to the deep adaptation model by just exploiting the deep features extracted from any one of the state-of-the-art deep models.

#### Ablation Study

In our method MA-PCA, there exist several hyper-parameters needed to be tuned. These hyper-parameters are mainly used to trade off different components of the proposed framework. We therefore, respectively, set these parameters into their extreme values to explore the importance of each component in MA-PCA. To this end, we set *S* = I to denote MA-PCA without target domain divergence information by MA-PCA_NTS and set γ_*i*_ = 1/*q* and $\gamma_{i}=\frac{\exp\left(-D\,i\,s\,t\,\left(X_{i}^{s},X\right)\right)}{\sum_{i}\exp\left(-D\,i\,s\,t\,\left(X_{i}^{s},X\right)\right)}$(*i* = 1,2,…,*q*)to, respectively, denote by MA-PCA_NSS and MA-PCA_NOS the case where MA-PCA weights each source model by mean components and measures its distance from the target domain, respectively.

The performance of these derived methods is evaluated on cross-dataset recognition tasks, and the performance results are shown in Table 2. It is easy to see from Table 2 that the performance of all derived methods is more or less degraded, and the performance of the MA-PCA_NTS method without target divergence constraints is slightly degraded. The performance of the MA-PCA_NSS method with an average weight on the source domain model decreases significantly; The performance of MA-PCA_NOS with the distance-weighted method for the source domain model is better than that of the MA-PCA_NSS method. However, the performance of these two derived methods is weaker than that of the MA-PCA optimization weighting method for the source domain model, which indicates that the proposed optimization mechanism for source domain model selection is effective. In addition, an interesting observation is that the overall recognition accuracy is below 60% when the multi-source domains are all from the SEED dataset. However, when the multi-source domain has the DEAP dataset, the overall recognition accuracy of all methods is close to 70%, even higher than 70%. It indicates that the diversity of source domains can improve the robustness and generalization of MA-PCA and its derived methods.

**TABLE 2:** Multi-source adaptation emotion recognition accuracies of derived methods as well as MA-PCA.

Method	{DEAP,SII,SIII} →SI	{DEAP,SI,SIII} →SII	{DEAP,SI,SII} →SIII	{SI,SII,SIII} →DEAP	{SI,SII} →DEAP	{SI,SIII} →DEAP
MA-PCA_NTS	72.81	70.52	68.57	55.90	54.20	55.81
MA-PCA_NSS	71.30	70.05	65.87	53.17	53.77	55.43
MA-PCA-NOS	71.61	69.86	66.28	53.49	54.23	55.66
MA-PCA	**73.47**	**71.12**	**68.85**	**56.33**	**54.46**	**55.87**

*Values in bold denote the best recognition rates. SI, session I; SII, session II; SIII, session III.*

## Conclusion

To deal with cross-subject/dataset EEG-based emotion recognition task, we proposed a multi-model adaptation method with possibilistic clustering assumption, i.e., MA-PCA, by exploiting the knowledge of the correlation between the source and target domains in the objective function. It suppresses the influence of noise/abnormal data and weakens the impact of model performance caused by the different distributions of training data and test data (i.e., source domain and target domain). In MA-PCA, the fuzzy entropy regularization term is used to weaken the influence of noisy data, and multi-domain adaptation learning method is used to establish a robust classification model to weaken the influence of different data distributions. The comprehensive experiments performed on two public datasets verify the effectiveness of MA-PCA in dealing with cross-subject/dataset emotion recognition. In most scenarios, our MA-PCA (or MA-PCA-VGG16/DAN) obtains the best results or comparable performance with respect to several representative baselines. Since the implementation of MA-PCA algorithm needs an iterative optimization procedure, how to improve the efficiency of MA-PCA and seek a more efficient algorithm would be an issue worthy of further study in our future research.

## Data Availability Statement

The original contributions presented in the study are included in the article/supplementary material, further inquiries can be directed to the corresponding author.

## Author Contributions

All authors listed have made a substantial, direct, and intellectual contribution to the work, and approved it for publication.

## Conflict of Interest

The authors declare that the research was conducted in the absence of any commercial or financial relationships that could be construed as a potential conflict of interest.

## Publisher’s Note

All claims expressed in this article are solely those of the authors and do not necessarily represent those of their affiliated organizations, or those of the publisher, the editors and the reviewers. Any product that may be evaluated in this article, or claim that may be made by its manufacturer, is not guaranteed or endorsed by the publisher.

## Footnotes

1 For each source domain, we train one SVM by using the corresponding labeled samples. Then, for each test instance *x*, the decision values from *p* SVM classifiers are converted into probability values by using the sigmoid function [i.e., g(t) = 1/(1 + exp(-t)]. Finally, we average the *p* probability values as the final prediction of the test instance *x*.
